# Serial transurethral cystometry: A novel method for longitudinal evaluation of reflex lower urinary tract function in adult female rats

**DOI:** 10.14814/phy2.15131

**Published:** 2022-01-03

**Authors:** Faiza Qureshi, Preston Kung, Wei Hou, William F. Collins, Sue Ann Sisto

**Affiliations:** ^1^ Health and Rehabilitation Sciences School of Health Technology & Management Stony Brook University Stony Brook New York USA; ^2^ Temple University Philadelphia Pennsylvania USA; ^3^ Department of Family Population & Preventive Medicine Stony Brook Medicine Stony Brook New York USA; ^4^ Department of Neurobiology and Behavior Stony Brook University Stony Brook New York USA; ^5^ Department of Rehabilitation Science School of Public Health and Health Professions University at Buffalo Buffalo New York USA; ^6^ Present address: Department of Anesthesiology Stony Brook University Stony Brook New York USA

**Keywords:** cystometry, rat, urinary bladder

## Abstract

**Aims:**

The aim of the study is to develop a minimally invasive method for longitudinal evaluation of lower urinary tract function that allows for simultaneous measurements of bladder pressure and external urethral sphincter (EUS) electromyographic (EMG) activity.

**Methods:**

To evaluate the reliability of serial transurethral cystometry (STUC), rats (*n* = 12) underwent three sessions of STUC, one session a week for 3 weeks. During each session, rats were anesthetized with ketamine–xylazine (90 mg/kg and 10 mg/kg), and micturition reflex data were acquired using transurethral cystometry and percutaneous recording of EUS (EMG) activity during continuous infusion of saline into the bladder. The reliability and consistency of the STUC method were assessed using intra‐class correlation (ICC) analysis and repeated measures ANOVA.

**Results:**

ICC values calculated from five successive events during the first micturition session indicate good to excellent reliability for measurements of peak bladder pressure, threshold bladder pressure, minimum bladder pressure, volume threshold, duration of EUS bursting, and number of EUS burst events. Across the three recording sessions no significant difference was observed in peak bladder pressure, threshold bladder pressure, minimum bladder pressure, volume threshold, number of EUS burst events, and duration of EUS bursting using repeated measures ANOVA.

**Conclusion:**

Serial transurethral cystometry under ketamine–xylazine anesthesia with simultaneous percutaneous EUS EMG recording is a novel, reliable, accurate, and minimally invasive method for quantitative assessment of lower urinary tract (LUT) function in adult female rats over extended periods of time.

## Graphical abstract

A minimally invasive and reliable method for evaluating lower urinary tract function in female rats that has the potential to be repeated over longer periods of time in models with neurological injury to asses and monitor lower urinary tract function.

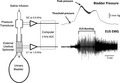

## 1 INTRODUCTION

In mammals, storage and elimination of urine requires the coordinated activity of sympathetic, parasympathetic, and somatic efferent motor pathways involving both segmental spinal reflexes (e.g., the activation of the external urethral sphincter [EUS] during bladder filling) and the well‐studied spino‐bulbo‐spinal reflexes that coordinate simultaneous bladder contraction and EUS relaxation during voiding (Fowler et al., 2008). Thus, it is not surprising that lower urinary tract (LUT) dysfunction is a common comorbidity in individuals with neurological disease or central nervous system injury (Phe et al., 2016; Podnar et al., 2006; Sakakibara, 2015; Schurch et al., 2015; Winge, 2015) that significantly impacts the health and quality of life of both patients and caregivers. Therefore, it is vital to investigate LUT dysfunction using approaches that include assessment of both the bladder and EUS function.

Rodents, most commonly rats, have been studied to assess storage and voiding functions using various forms of cystometry (Andersson et al., 2011). Researchers have used either acute suprapubic (Hubscher et al., 2016; Kruse et al., 1993; Leung et al., 2007; Mitsui et al., 2014; Ward et al., 2013; Yoshiyama et al., 1999), chronic suprapubic (Schneider et al., 2015), or acute transurethral (Lee et al., 2013; Pikov & Wrathall, 2001) approaches to perform cystometries either under anesthesia (Kruse et al., 1993; Ward et al., 2013) or in awake/restrained rats (Hubscher et al., 2016; Lee et al., 2013; Leung et al., 2007; Mitsui et al., 2014; Pikov & Wrathall, 2001; Schneider et al., 2015; Yoshiyama et al., 1999). Few studies have evaluated simultaneous bladder and EUS function (D'Amico et al., 2011; Kruse et al., 1993; Lee et al., 2013; Leung et al., 2007; Pikov & Wrathall, 2001; Schneider et al., 2015). LaPallo et al., 2014, 2017 measured only chronic EUS function without simultaneous bladder pressure recordings while other investigators measured only bladder function without simultaneous EUS electromyographic (EMG) recording (Hubscher et al., 2016; Mitsui et al., 2014; Ward et al., 2013; Yoshiyama et al., 1999).

The goal of the present study is to develop a less invasive method for longitudinal evaluation of LUT function that allows for simultaneous measurements of bladder pressure and EUS EMG activity. To this end, we have implemented this approach utilizing transurethral cystometry for measuring bladder pressure combined with percutaneous recording of EMG activity of EUS muscle while infusing saline into the bladder to elicit micturition events. The combination of transurethral cystometry and percutaneous EUS EMG recording is substantially less invasive compared to the implanted methods and has the potential to be repeated in the same animal over an extended period of time. Thus, serial transurethral cystometry (STUC) can be used to monitor LUT function in individual subjects before and after experimental manipulations and to assess the efficacy of treatments and interventions. In the present report, we demonstrate in an adult rat model that STUC is a reliable and accurate method for longitudinal investigation of LUT function.

## 2 MATERIALS AND METHODS

### 2.1 Animal subjects

Twelve (12) female Sprague Dawley rats (225–250 g; Taconic Biosciences) were used in this study. Rats were housed individually in ventilated cages with food and water ad libitum in a humidity and temperature‐controlled room with a 12‐h light/dark cycle. Institutional Animal Care and Use Committee of Stony Brook University approved all procedures.

### 2.2 Experimental design

To evaluate the reliability of STUC, rats underwent three sessions of STUC. For most animals, the time between recording sessions was 5–9 days (mean = 6.4, median 7). However, in some cases the time between recording sessions was as short as 1–2 days (2 rats) or as long as 14 days (1 rat). During each STUC session, rats were anesthetized with ketamine–xylazine, and micturition reflex data were acquired using transurethral cystometry and percutaneous recording of EUS (EMG) activity during continuous infusion of (room temperature) saline into the bladder. The reliability and consistency of the STUC method were assessed using intra‐class correlation analysis (during the first session) and repeated measures ANOVAs (between the three sessions).

### 2.3 Transurethral cystometry with percutaneous EUS EMG recording

#### 2.3.1 Anesthesia

Initially a mixture was prepared from ketamine (100 mg/ml) and xylazine (20 mg/ml). The rats were then anesthetized with a dose of ketamine (90 mg/kg) and xylazine (10 mg/kg) i.p. to achieve areflexia (lack of withdrawal to pinching of the forepaw). Supplemental doses were given as i.p. (ketamine 3.2 mg and xylazine 0.36 mg) only in five recording sessions. These supplemental doses were given when it was observed that the animal was light as monitored by rapid respiration, body movements, and paw pinch. Body temperature was maintained at 37ºC using a heating pad.

#### 2.3.2 Data acquisition

**FIGURE 1:**
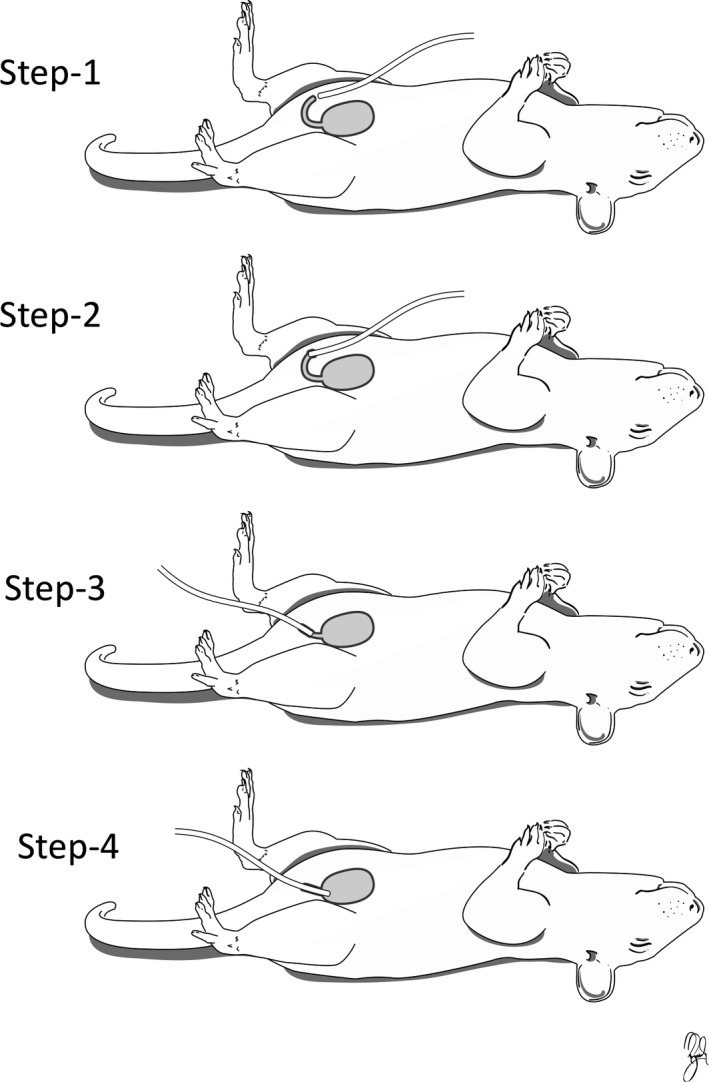
Steps of transurethral cystometry. (1) PE‐50 catheter tip facing caudally and close to urethral orifice. (2) Catheter entering the urethral orifice in a caudal direction, in a plane parallel to the body of the animal. (3) & (4) Catheter rotated 180^°^ to enter the bladder

**FIGURE 2:**
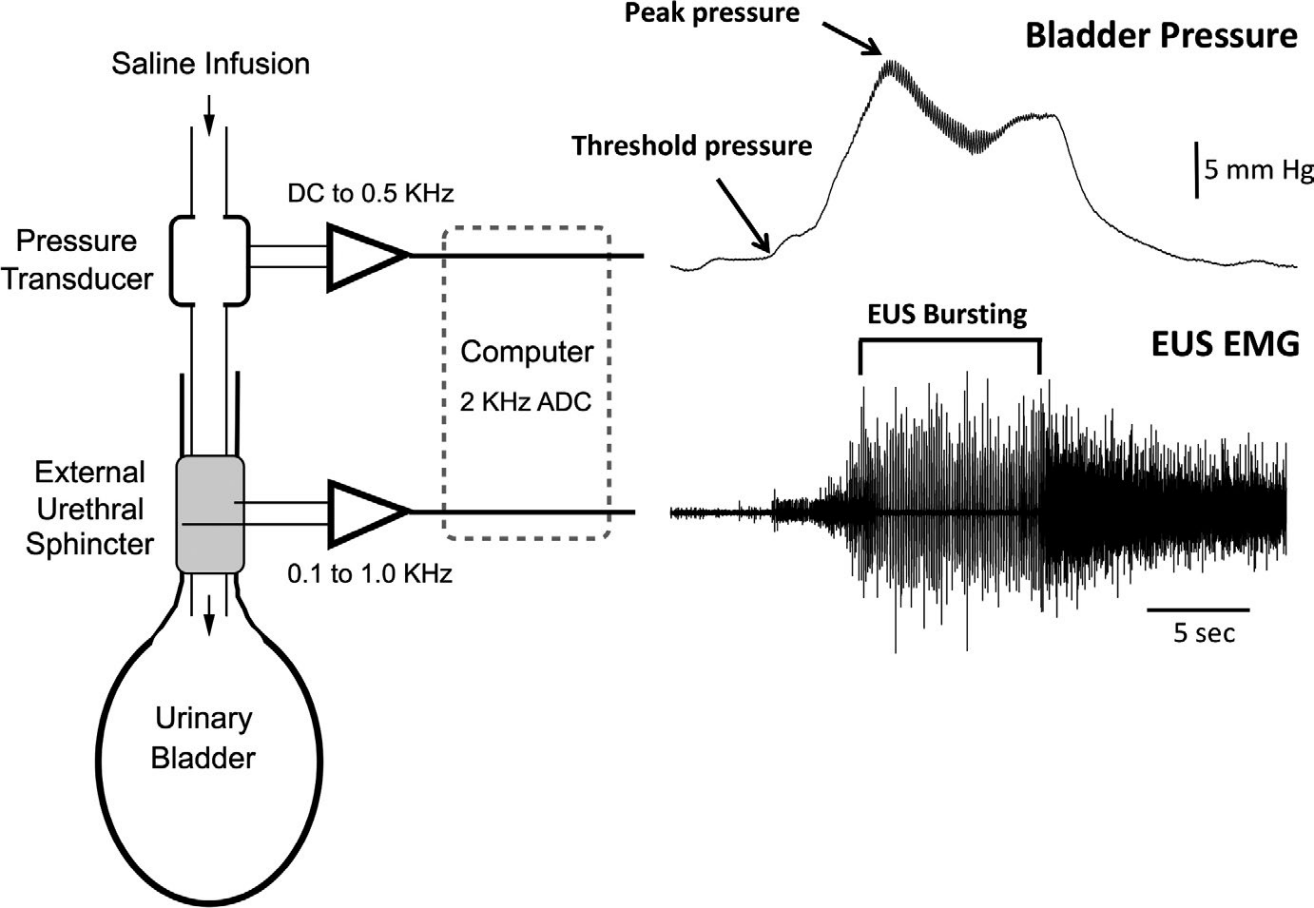
Transurethral cystometry (TUC) set up. PE‐50 catheter is introduced through the urethra into the bladder. The catheter is connected in series to a pressure transducer to record bladder pressure recordings and to a syringe pump to deliver saline at a continuous rate. EUS EMG recordings were acquired by inserting two fine wire electrodes bilaterally through the perineal skin and under the ventral aspect of the pubic symphysis

With the animal in a supine position, bladder was expressed manually and a sterile catheter (PE‐50, AM systems.) was placed through the urethra into the bladder. Catheter was marked at two sites from the tip (2 cm and 3 cm). The catheter was advanced into the urethral opening in a caudal direction to the first mark and then rotated 180^°^ toward the bladder up to the second mark (Figure 1). In a few cases where there was difficulty of passing the catheter, sterilized vaseline was used at the tip of the catheter as a lubricant. The catheter was then connected in series to a pressure transducer (to record bladder pressure) and to a 60 cc syringe attached to a syringe pump (Harvard Apparatus, Holliston, MA) to infuse saline into the bladder and with a pump rate of 0.088 ml/min. EUS EMG recordings were made by inserting two sterile fine wire electrodes (A‐M Systems; 50 μm insulated stainless steel) percutaneously into or near the EUS muscle. This was achieved by putting each electrode through a 30‐gauge needle and bending a short length of exposed wire back to form a hook. Each needle/electrode was inserted through the perineal skin (~3–5 mm on either side of the urethral meatus) and advanced under the pubic symphysis to the vicinity of the EUS (insertion depth 13 mm). The needle was then withdrawn gently, leaving the electrode in place. Sterile saline at room temperature was infused (0.088 ml/min) through the catheter into the bladder to evoke repetitive micturition events during which bladder pressure and EUS EMG were recorded (Figure 2). The position of the electrodes was confirmed in two ways: 1. by evoking a low threshold response to a gentle pressure on the abdomen and 2. EMG activity associated with the micturition events. Recordings of bladder and EUS were continued up to 2 h. Bladder catheter and EUS electrodes were then gently removed. Observation followed to be sure that there is no bleeding from urethra or EUS. Necropsy was not required as the electrodes were not implanted and were removed at the end of each recording session. The rat was placed back to its home cage and the home cage was placed on a heating pad. The rat was monitored there until it fully recovered from anesthesia. Then it was moved back to the animal facility.

The EUS EMG signal was amplified (x1K) and filtered (10 Hz–1 KHz) through a differential AC preamplifier (A‐M Systems Model 1700). The BP signal was amplified through a PM‐1000 transducer amplifier (CWE Inc.) and filtered (DC to 0.5 kHz). Data were acquired at 2 KHz using a PowerLab 16/35 and LabChart 8.0 software (AD Instruments) and analyzed using custom procedures in Igor Pro 6.3 (WaveMetrics). Each recording session lasted approximately 2 h. In recording sessions, where supplemental doses were given, there was no change in the average urodynamic parameters.

### 2.4 Data analysis

In most cases a stable pattern of repetitive rhythmic voiding cycles was observed approximately 30–45 mins from the administration of anesthesia. Features of micturition events like peak pressure and inter‐contraction interval, etc. were quantified during this stable period. Basic urodynamic feature measurements were made from bladder pressure (BP) and rectified EUS EMG records from five micturition events, smoothed by software resampling to achieve effective sampling rates of 0.1 KHz. BP records were corrected for any offset pressure due to resistance of the catheter by subtracting the pressure recorded at the beginning of infusion when the bladder was relatively empty. Measurements from five successive micturition events were averaged, reflecting the mean measurement from one animal.

Threshold bladder pressure was measured as the pressure at the beginning of active bladder contraction when there was an abrupt increase in the slope of the BP record. Peak bladder pressure was measured as the difference between peak bladder pressure during contraction and the lowest bladder pressure after a void. EUS bursting was quantified by measuring the duration of EUS bursting (time from the onset of the first EUS EMG quiet period to the end of the last EUS EMG quiet period) and counting the number of EUS burst events (the number of EUS EMG peaks with amplitudes greater than 2× standard deviation above baseline). Volume threshold was calculated as the product of intercontraction interval (ICI) × infusion rate.

### 2.5 Statistical analysis

The reliability of feature measurements acquired with the transurethral cystometry approach was assessed by calculating the intra‐class correlation coefficient ICC‐3,k (Koo & Li, 2016) for each feature acquired from five successive micturition events during the first recording session in each rat. The calculations were performed in R using the ICC procedure in the “psych” package (version 1.8.4), and both ICC values and 95% confidence intervals are reported. The reliability of the measurements across transurethral cystometry recording sessions was assessed by performing repeated measure ANOVAs (aov procedure in R) for each feature. A criterion level of *p* < 0.05 was used for the determination of statistical significance.

## 3 RESULTS

To assess the reliability of transurethral cystometry *within* a recording session, intra‐class coefficients (ICC‐3) were calculated for multiple feature measurements from five successive micturition events during the first recording session. The ICC values indicate good to excellent reliability for measurements of peak bladder pressure (0.86), threshold bladder pressure (0.87), minimum bladder pressure (0.96), volume threshold (0.97), duration of EUS bursting (0.91), and number of EUS burst events (0.93).

**FIGURE 3:**
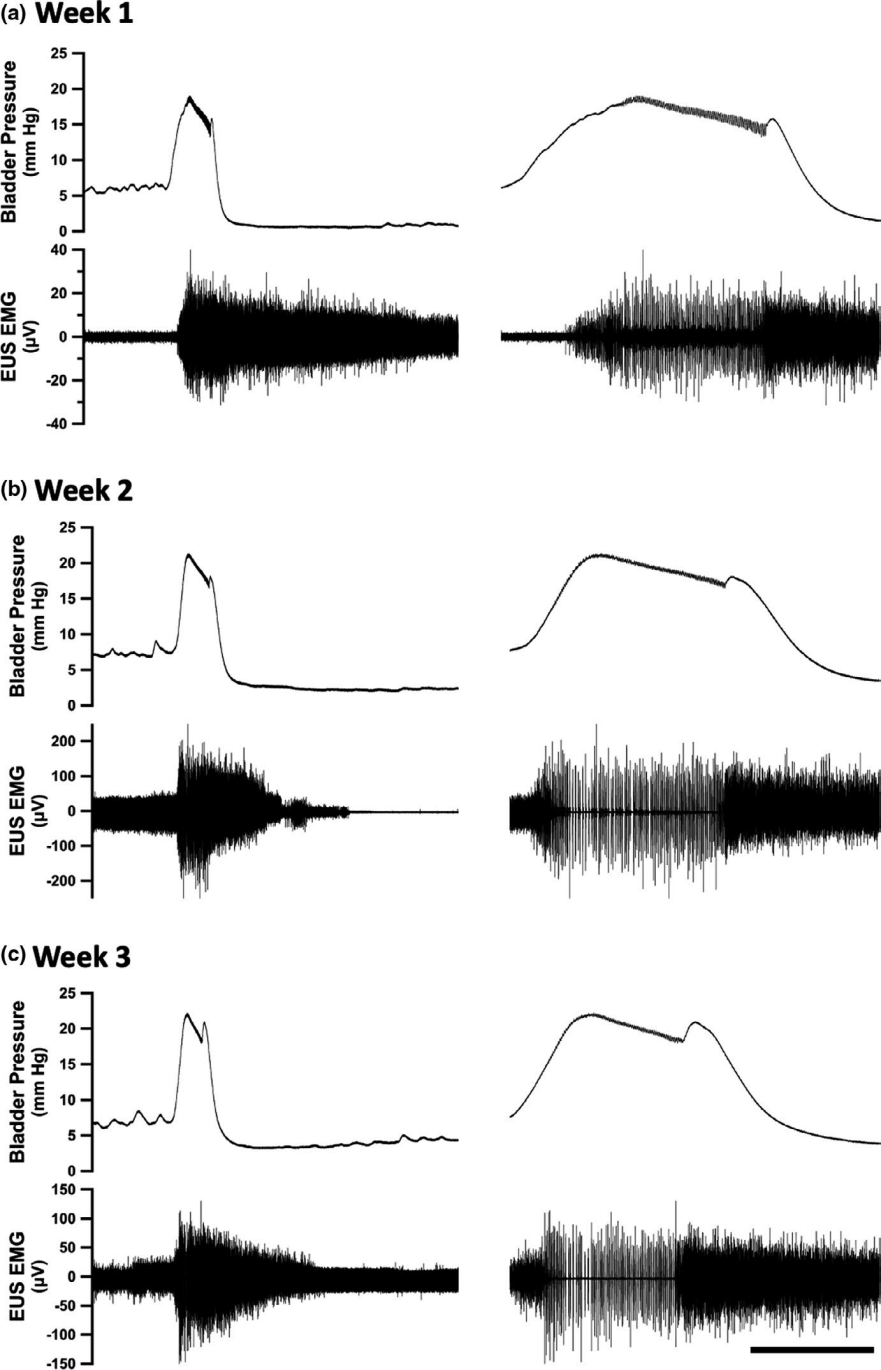
Serial Transurethral Cystometry: Example Recordings. Bladder pressure (top) and EUS EMG recordings (bottom) acquired during single micturition events from the same rat during separate transurethral recording sessions over a three‐week time period; (a) week1, (b) week 2, (c) week 3. Expanded records are shown in the column on the right. Calibration bar indicates 60 and 10 s (left and right records, respectively)

**FIGURE 4:**
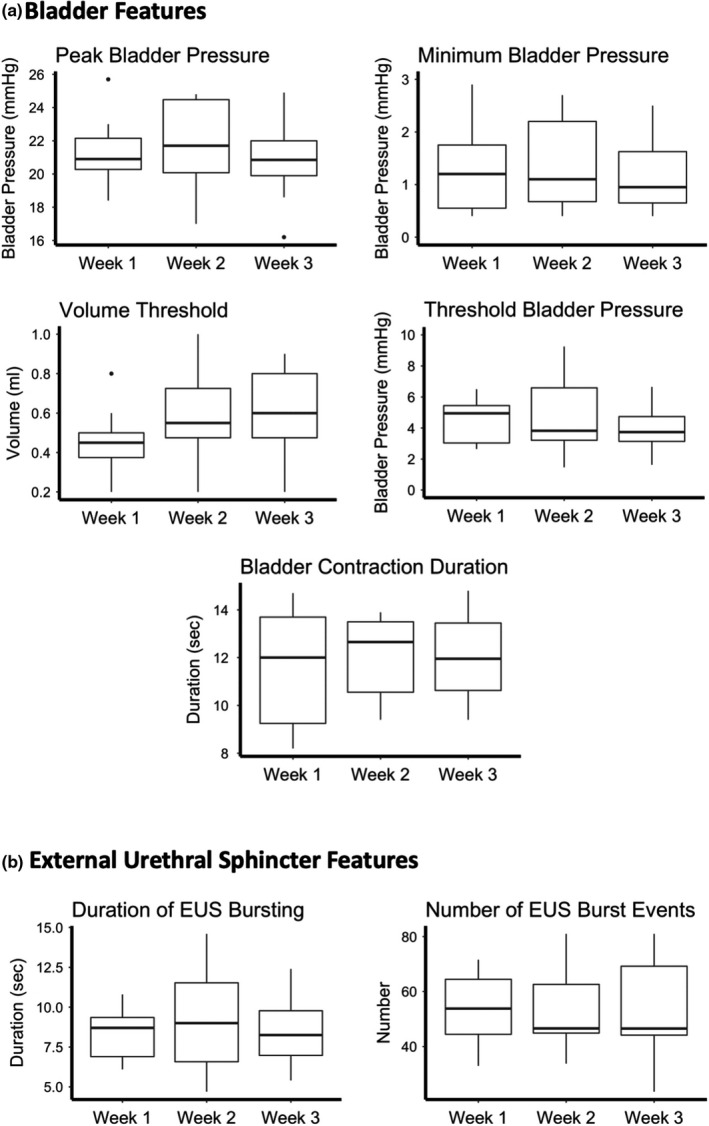
Serial transurethral cystometry: summary graphs. Box and whisker plots of bladder pressure (a) and EUS EMG feature measurements (b) acquired during micturition events recorded using serial cystometry over a 3‐week period. During each recording session, five successive micturition events were averaged. No statistically significant differences between the three recording sessions were observed (repeated measures ANOVA) in all variables

The reliability of STUC *across* sessions was assessed by analyzing feature measurements acquired from five successive micturition events in each of all three recording sessions (Figure 3). Using repeated measure ANOVAs, no significant difference was observed in peak bladder pressure, threshold bladder pressure, minimum bladder pressure, threshold volume, number of EUS burst events, and duration of EUS bursting across recording sessions (Table 2, Figure 4).

## 4 DISCUSSION

The present study demonstrates that STUC under ketamine–xylazine anesthesia is an accurate and reliable protocol and offers numerous advantages for longitudinal study of reflex lower urinary tract function. Transurethral catheterization and percutaneous placement of EUS EMG electrodes are straightforward procedures in adult female rats that allow for accurate measurement of bladder pressure and EUS activity during continuous flow cystometry. The protocol produces consistent results within a recording session (ICC‐3, Table 1) as well as across recording sessions (repeated measures ANOVA, Table 2). It is less invasive than methods that use chronic implantation techniques, resulting in little trauma to the bladder and urethra and reduced complications compared to chronic catheter and electrode implantation (e.g., inflammation, urinary tract infection, urolith, bleeding, and catheter blockade) (Morikawa et al., 1989; Yaksh et al., 1986). No evidence of inflammation, catheter blockade, or internal bleeding was observed. We used a very small caliber transurethral catheter (PE‐50). Catheter insertion and removal were performed very gently and the rat was observed to make sure that there was no bleeding from the urethra. A histology section of the urethral or bladder tissue was not possible because the rats were not sacrificed after the last recording session. Rather, they were used for additional studies. At the end of this study each rat underwent a separate terminal transvesical cystometry experiment. This gave us an opportunity to further verify that there was no injury to the bladder and EUS. The STUC protocol is well tolerated and affords maximal flexibility in experimental design and execution. The approach can be used with few limitations at any time during an extended study and does not require complicated surgical procedures to implant chronic instrumentation. Furthermore, transurethral catheterization is a commonly performed procedure in humans and is an appropriate experimental model for translational studies. Potential limitations of transurethral cystometry include partial obstruction of the urethra and particularly in smaller subjects, which could interfere with voiding leading to increased residual volume. We minimize the risk of injury using fine wire electrodes in the EUS muscle that straightened while pulling, reducing its potential for injury and the bursting events were unaffected in our subsequent recording sessions. There was no apparent injury through observation of the EUS muscle prior to the subsequent terminal trans‐vesical experiments. Another potential limitation is that the placement of electrodes can be different each time that may reflect in variable sustained post‐void tonic activity. We were aware of this limitation and our focus was to study EUS bursting that is less sensitive to placement of EUS electrodes. Also, species‐ and gender‐dependent differences in the anatomical characteristics of the urethra will dictate whether or not the transurethral procedure is feasible for animals other than adult female rats. Our method is a continuous flow cystometry model that does not allow for infusion pump to be stopped to empty bladder and restart to fill again, a method that is required to measure residual volume and voiding efficiency. The STUC method could be modified to measure residual volume by stopping the pump immediately after a micturition event and emptying the bladder by reverse flow through the transurethral catheter. This was not performed in the present study. The use of anesthesia during the recording session has both advantages and limitations. It eliminates the need to restrain subjects and the associated stress on the subject (Morikawa et al., 1990). Furthermore, the use of anesthesia minimizes possible recording artifacts caused by motor activity during cystometry (Leung et al., 2007). However, possible effects of the anesthesia on micturition reflexes and broader experimental outcomes must be considered. When comparing between anesthetics, urethane produces bladder pressure profiles similar to conscious and decerebrate preparations and has a long half‐life. However, its use is limited to only non‐survivable procedures attributed to its carcinogenic and mutagenic effects in rodents (Fraser et al., 2020). In the present study, rats were anesthetized with a mixture of ketamine, an NMDA receptor blocker, and xylazine, an adrenergic α2 receptor agonist with analgesic properties. Although NMDA receptor blockade has been shown to inhibit reflex micturition (Yoshiyama et al., 1993), urodynamic features of reflex micturition in the present study are comparable to that in other studies (Table 3). The only differences noted were (1) the longer duration of EUS bursting, a feature characteristic of rodent models and (2) a low threshold pressure. It is interesting to note that ketamine is used during transurethral cystometry in pediatrics to reduce movement artifacts in uncooperative children without affecting the urodynamic properties (Thevaraja et al., 2013). Also, the analgesic properties of xylazine minimize pain, which may be a complicating factor during recordings from an awake animal shortly after anesthesia with an implanted suprapubic catheter. There have been reports of ketamine‐induced cystitis related to its chronic abuse and recreational use (Ho et al., 2010; Shahani et al., 2007). In our experiments, we used intraperitoneal dose of ketamine 90 mg/kg and xylazine 10 mg/kg once a week. The reported half‐life of ketamine is 2 h and for xylazine is 1 h in young Sprague Dawley rats. Clearance for ketamine is 2.5 days and for xylazine it is 4–5 days (Veilleux‐Lemieux et al., 2013). This makes using ketamine and xylazine a safe drug for repeated use with at least a gap of 5 days in between administrations. For most subjects, the time between recording sessions was 5–9 days (mean 6.4, median 7) and therefore it was safe to use it without potential complications. While the recording sessions in the present study were conducted during a period of 3 weeks, this method could be used for repeated assessment of LUT function over the extended time course of a chronic experiment with expected reliability.

**TABLE 1:** Intraclass Correlation Coefficients (ICC‐3) were calculated over 5 successive micturition events during the first recording session (ICC 3 column) to assess the reliability of transurethral cystometry measurements

	ICC−3 Week 1 (95% CI)
Peak bladder pressure (mmHg)	0.86 (0.67, 0.95)
Threshold bladder pressure (mmHg)	0.87 (0.71, 0.95)
Minimum bladder pressure (mmHg)	0.83 (0.62, 0.94)
Volume threshold (ml)	0.97 (0.93, 0.99)
Burst duration (s)	0.91 (0.80, 0.97)
Burst number	0.94 (0.86, 0.98)

ICC values < 0.5 are indicative of poor reliability, values 0.5–0.75 indicate moderate reliability, values 0.75–0.9 indicate good reliability and values >0.9 indicate excellent reliability. All feature measurements show good to excellent reliability.

**TABLE 2:** Repeated ANOVA for serial cystometry measurements across the three sessions (week1, week2, and week3) did not show any significant differences of means

	ANOVA			
	Week 1 mean (SD)	Week 2 mean (SD)	Week 3 mean (SD)	*p* value
Peak bladder pressure (mmHg)	21.4 (1.89)	21.8 (2.57)	20.8 (2.21)	0.176
Threshold bladder pressure (mmHg)	4.43 (1.39)	4.87 (2.65)	3.85 (1.39)	0.431
Minimum bladder pressure (mmHg)	1.28 (0.82)	1.44 (0.87)	1.27 (0.73)	0.821
Volume threshold (ml)	0.48 (0.18)	0.64 (0.24)	0.63 (0.22)	0.136
Burst duration (s)	8.33 (1.59)	9.30 (3.47)	8.72 (2.32)	0.558
Burst number	53.9 (13.83)	53.3 (16.30)	52.7 (16.72)	0.965

Each mean is an average of 5 successive micturition events from each rat. Units in the left column apply to values in the Week 1, Week 2 and Week 3 columns.

**TABLE 3:** Comparison of means (SD) of week 1 serial cystometry measurements (*n* = 12) with cystometry experiments reported in the literature

Reliability analysis (Week 1)––Comparison with Literature		
	Week 1	References
Peak bladder pressure (mmHg)	21.4 (1.8)	25.7(2) (Yoshiyama et al., 1999), 25.4 (7.7) (Pikov & Wrathall, 2001), 20.5 (2) (Kruse et al., 1993)
Threshold bladder pressure (mmHg)	4.43 (1.3)	9.5 (1) (Yoshiyama et al., 1999), 8.0 (2) (Kruse et al., 1993)
Burst duration (s)	8.33 (1.5)	3.7 (0.45) (Langdale & Grill, 2016), 2.4 (0.78) (D'Amico et al., 2011)

We did not do a comparison of STUC with terminal transvesical method because the terminal transvesical cystometry was performed after a significant delay and the animals were utilized as a part of another study. We, therefore, took the opportunity to compare our method to already published results in the literature (Table 3). Use of a small caliber catheter (PE‐50) helped avoiding any obstructive effects like high bladder pressures as have been reported by some (Smith et al., 2008). Bladder pressures recorded in STUC were comparable to others reported in the literature (Table 3).

## 5 CONCLUSION

Serial transurethral cystometry under ketamine–xylazine anesthesia with simultaneous percutaneous EUS EMG recording is a, reliable, accurate, and less invasive method for quantitative assessment of LUT function in adult female rats over extended periods of time (e.g., weeks to months). This method can be considered in chronic experiments, models of pathology, and neurological injury in which other approaches to longitudinal monitoring of LUT function may be impractical.

## CONFLICT OF INTEREST

No conflict of interest, financial or otherwise, are declared by the authors.

## AUTHOR CONTRIBUTIONS

**Faiza Qureshi** (First author/corresponding author): design and execution of experiments, data analysis, interpretation of results, writing and revision of the manuscript, and final approval of the manuscript. **Preston Kung** (Co‐author): acquisition and analysis of data, and revision and final approval of the manuscript. **Wei Hou** (Co‐author): statistical analysis of data, and revision and final approval of the manuscript. **William F. Collins III** (Senior Co‐author): design of experiments, interpretation of results, and revision and final approval of the manuscript. **Sue Ann Sisto** (Senior Co‐author): design of experiments, interpretation of results, and revision and final approval of the manuscript.
